# CuFeAl Nanocomposite Catalysts for Coal Combustion in Fluidized Bed

**DOI:** 10.3390/nano10051002

**Published:** 2020-05-24

**Authors:** Aleksandr V. Fedorov, Nikolay A. Yazykov, Olga A. Bulavchenko, Andrey A. Saraev, Vasily V. Kaichev, Vadim A. Yakovlev

**Affiliations:** Boreskov Institute of Catalysis, Lavrentev Ave., 5, 630090 Novosibirsk, Russia; yazykov@catalysis.ru (N.A.Y.); isizy@catalysis.ru (O.A.B.); asaraev@catalysis.ru (A.A.S.); vvk@catalysis.ru (V.V.K.); yakovlev@catalysis.ru (V.A.Y.)

**Keywords:** catalytic combustion, fluidized bed, CO oxidation, oil-drop granulation, iron oxide, copper oxide

## Abstract

A method of oil-drop granulation was suggested for the preparation of spherical CuFeAl nanocomposite catalysts. The catalysts were characterized by a set of physicochemical methods (X-ray diffraction, temperature-programmed reduction by H_2_, low-temperature nitrogen adsorption, crushing strength) and tested in the oxidation of CO and burning of brown coal in a fluidized bed. It was found that the catalysts have high mechanical strength (16.2 MPa), and their catalytic properties in the oxidation of CO are comparable to the characteristics of industrial Cr-containing catalysts. It was shown that the addition of pseudoboehmite at the stage of drop formation contributes to the production of uniform spherical high-strength granules and facilitates the stabilization of the phase state of the active component. The use of CuFeAl nanocomposite catalysts for the burning of brown coal provides a low emission of CO (600 ppm) and NO*_x_* (220 ppm) and a high degree of coal burnout (95%), which are close to those of the industrial Cr-containing catalysts (emission of CO is 700 ppm, NO*_x_*—230 ppm, and degree of coal burnout is 95%).

## 1. Introduction

The method of burning organic materials in a fluidized catalyst bed has several advantages over traditional methods of combustion [1,2,3,4,5]. In particular, the lower temperature of the process allows one to reduce the requirements for construction materials and to prevent side endothermic reactions leading to the formation of toxic nitrogen oxides (NO*_x_*). Fluidized bed catalytic combustion provides higher heat densities in the combustion zone due to the localization of oxidation reactions on the catalyst surface, which in turn reduces the size, weight, and capital cost of industrial catalytic installations [6,7].

The catalytic combustion of solid fuels in a fluidized bed imposes a number of requirements for industrial catalysts:

1. High catalytic activity;
2. High mechanical strength and attrition resistance;
3. Spherical shape and narrow distribution of the granule size;
4. Catalytic stability throughout the catalyst lifetime.

The high activity of catalysts is required primarily due to the need to achieve higher levels of fuel combustion efficiency, as well as low levels of harmful gas emissions (CO, CH_4_, and NO*_x_*). The high mechanical strength is required due to the severe operating conditions in a fluidized bed [8]. The spherical shape of granules helps to reduce the catalyst waste due to attrition. The catalytic stability requires high sustainability of the catalyst not only to process the conditions (high temperature, redox environment), but also catalytic poisons (sulfur oxides, alkaline metals, etc.) [2,9,10,11].

The oil-drop granulation method allows the obtaining of spherical Al_2_O_3_ granules, which can be used as a support for the catalysts operating in a fluidized bed [12,13,14]. The catalysts that satisfy the requirements above and that are commonly used for fluidized bed combustion are Cr-containing catalysts supported on alumina granules [15]. However, a significant drawback of these catalysts is the presence of toxic chromium, which inevitably pollutes the atmosphere as a result of attrition [16]. Iron oxide can be a good alternative to the chromium-containing active component, since catalysts based on Fe_2_O_3_ have a significant activity in deep oxidation [17,18,19], less toxicity, and a significantly lower cost. Moreover, as we have previously shown, the addition of aluminum and copper oxides can substantially increase the catalytic activity of iron oxide-based catalysts [20,21,22,23,24]. In addition, the introduction of alumina into Fe_2_O_3_ enhances the dispersion of iron oxide particles and, as a result, increases the activity of the catalyst [24]. In turn, the addition of copper lowers the reduction temperature of iron oxide and leads to the formation of copper-containing phases, thereby increasing the activity of the catalyst in the oxidation of CO [22,24]. It was also shown that a nanodispersed CuFeAl catalyst is highly active in the oxidation of CO [20,25]. In the present work, the powder of the CuFeAl catalyst was used as the active component for the preparation of spherical granules of the CuFeAl nanocomposite catalyst.

The spherical granules of the CuFeAl nanocomposite catalyst were prepared by the oil-drop granulation method. The active component was added at the stage of preparing the pseudo-sol, followed by drop formation of the resulting suspension. The proposed approach can significantly increase the content of the active component and, as a result, the activity of a synthesized catalyst. This paper presents the results of a study of the physicochemical and catalytic properties of the CuFeAl nanocomposite catalyst. The catalyst was tested in the burning of brown coal in a fluidized bed in comparison with a common chromium-containing industrial catalyst.

## 2. Materials and Methods

### 2.1. Catalyst Preparation

The powdered CuFeAl catalysts were prepared by the melting of copper, iron, and aluminum nitrates Cu(NO_3_)_2_·3H_2_O (98.0%, ReaHim Ltd., Moscow, Russia), Fe(NO_3_)_3_·9H_2_O (99.0%, ReaHim Ltd., Moscow, Russia), and Al(NO_3_)3·9H_2_O (99.0%, ReaHim Ltd., Moscow, Russia). To prepare 10 g of the CuFeAl nanocomposite catalyst, the nitrates (1.55 g of Cu(NO_3_)_2_·3H_2_O, 39.82 g of Fe(NO_3_)_3_·9H_2_O, 12.70 g of Al(NO_3_)_3_·9H_2_O) were mixed; the mixture was heated to give a homogeneous melt of hydrate salts (≈130 °C) and then was kept at a temperature of 200 °C, forming a solid precipitate. Finally, the resulting dry powder was calcined at 450 °C for 1 h in air using a furnace (WiseTherm FX-03, Daihan Scientific Co. Ltd., Seoul, South Korea). The powdered catalysts consisted of oxides of the following calculating composition: 5.0 wt% CuO, 77.9 wt% Fe_2_O_3_, and 17.1 wt% Al_2_O_3_.

**Figure 1:**
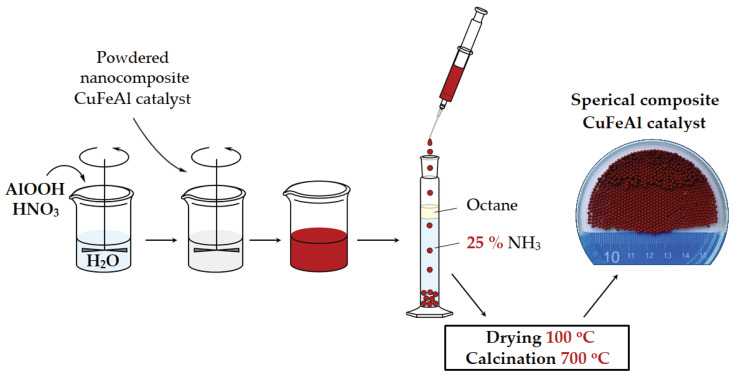
Scheme of the spherical CuFeAl nanocomposite catalyst preparation.

The spherical CuFeAl nanocomposite catalysts were prepared by the oil-drop granulation method. For this, 2M HNO_3_ was added dropwise under stirring for 10 min using a mechanical stirrer (2000 rpm, Eurostar digital, IKA-Werke GmbH, Staufen im Breisgau, Germany) to the suspension of aluminum hydroxide AlOOH (76.0 wt% Al_2_O_3_, <0.002 wt% Na_2_O, Pural SCF, Sasol Germany GmbH, Hamburg, Germany) in distilled water. The obtained suspension was diluted with distilled water and then was settled. A portion of the CuFeAl catalyst was added to the resulting suspension, and the obtained slurry was thoroughly mixed using the mechanical stirrer (2000 rpm, 10 min). Formation of spherical granules was carried out by generating sol droplets (3 mm diameter) with an adjutage, passing-through a paraffin oil layer, followed by gelating and aging in an ammonia layer. Resultant granules were slowly dried in an argon environment for 10 h at room temperature followed by 1 h at 100 °C and then calcined in air at 400 °C for 1 h and at 700 °C for 1 h with a heating rate of 15 °C/min. The sample was cooled to room temperature in a calcining furnace (5–10 °C/min). Figure 1 shows a scheme of the preparation of the spherical CuFeAl nanocomposite catalysts. Table 1 shows the conditions used for the preparation of the catalysts by drop granulation.

To prepare a 2M HNO_3_ solution, we used 70% nitric acid (ReaHim Ltd., Russia) and 25% ammonia solution (ReaHim Ltd., Russia). As the paraffin oil layer, n-octane CH_3_(CH_2_)_6_CH_3_ (95.0%, “ReaHim” Ltd., Moscow, Russia) was used.

### 2.2. X-ray Diffraction

The phase composition of the CuFeAl nanocomposite catalysts was studied by X-ray diffraction using a D8 Advance X-ray diffractometer (Bruker, Ettlingen, Germany) equipped with a Lynxeye linear detector. The monochromatic Cu Kα radiation (*λ* = 1.5418 Å) was applied for analysis. The XRD patterns were obtained in the 2θ range from 15° to 80° with a step of 0.05°. The coherent scattering region (average crystallite size) was determined using the Scherrer equation from the full width half maximum of the diffraction peaks. The coherent scattering region of hematite was calculated from the 140 reflection.

### 2.3. Specific Surface Area Analysis

The catalysts were analyzed by low-temperature nitrogen porosimetry using an automated volumetric adsorption analyzer ASAP 2400 (Micromeritics Instrument Corp., Norcross, GA, USA). Before the recording of the nitrogen adsorption isotherms, the samples were outgassed at 150 °C and a pressure of 0.13 Pa for 4 h. The initial branch of the N_2_ adsorption isotherm in a range of P/P_0_ from 0.05 to 0.2 was used to calculate the specific surface area (SSA) by the Brunauer–Emmett–Teller method.

### 2.4. Crushing Strength

The crushing strength of the spherical alumina granules was measured using a commercial strength tester (MP-9S, Novosibirsk Instrument-Building Plant, Novosibirsk, Russia). The spheres were loaded diametrically and then pressed between two rigid platens at a constant crosshead speed of 0.1 mm/s. The load at which the fracture of the sphere occurred was recorded as the crushing strength (*F*). The fracture stress (*σ*) was used to describe the mechanical strength of porous materials. It is known that *σ* does not depend on the shape and size (tablets, spheres, etc.) of pellets in contrast to the crushing strength. For spherical pellets, Hiramatsu and Oka showed [26] that the fracture stress is estimated from the crushing strength as follows:

$$\sigma =\frac{2.8F}{\pi d^{2}},$$

where *d* is the granule diameter, and *F* is the crushing strength. The number of measurements was 100 granules.

### 2.5. Temperature-Programmed Reduction

The temperature-programmed reduction of TPR-H_2_ was performed using a Chemosorb analyzer (Modern Laboratory Equipment, Novosibirsk, Russia). For all experiments, a sample (0.1 g) was first placed in a U-tube quartz reactor. The sample was then heated up to 900 °C with a constant heating rate of 8 °C/min using a 10% H_2_/Ar flow (30 mL/min) as a reducing agent. The hydrogen consumption was measured with a thermal conductivity detector.

### 2.6. Catalytic Activity Tests

The catalytic tests were performed using a catalytic setup with a flow-fixed bed reactor. A powdered sample with a particle size of 0.2–0.5 mm was loosely packed into a tubular U-shaped quartz reactor with a 3.8-mm inner diameter and a 10-mm length. The catalyst volume was 113 mm^3^. During the experiments, a gas mixture containing 20 vol.% O_2_ and 80 vol.% He was passed through the reactor with a constant flow rate of 30 cm^3^/min and CO was injected into the gas mixture in the pulse mode (0.1 cm^3^ pulse-1 for 0.75 s). The catalyst was heated from 25 to 350 °C with a constant rate (30 °C/min). Simultaneously, the concentrations of CO were monitored at the reactor outlet with a thermal conductivity detector and the catalytic activity was determined as the temperature of the 50%-CO conversion (T_50_). The catalytic activity of synthesized catalysts was compared with a widely used commercial catalyst—Cu*_x_*Mg*_1−x_*Cr_2_O_4_/Al_2_O_3_ (ForAlumina Ltd., Yarovoye, Russia). The commercial catalyst is the mix of copper oxide and magnesium chromites deposited on spherical alumina granules. Composition: 1.7% of CuO, 3.6% of MgO, 17.0% of Cr_2_O_3_, and the rest of Al_2_O_3_. The specific surface area for the commercial catalyst is 120 m^2^/g.

### 2.7. Coal Combustion in a Fluidized Bed of a Catalyst

**Figure 2:**
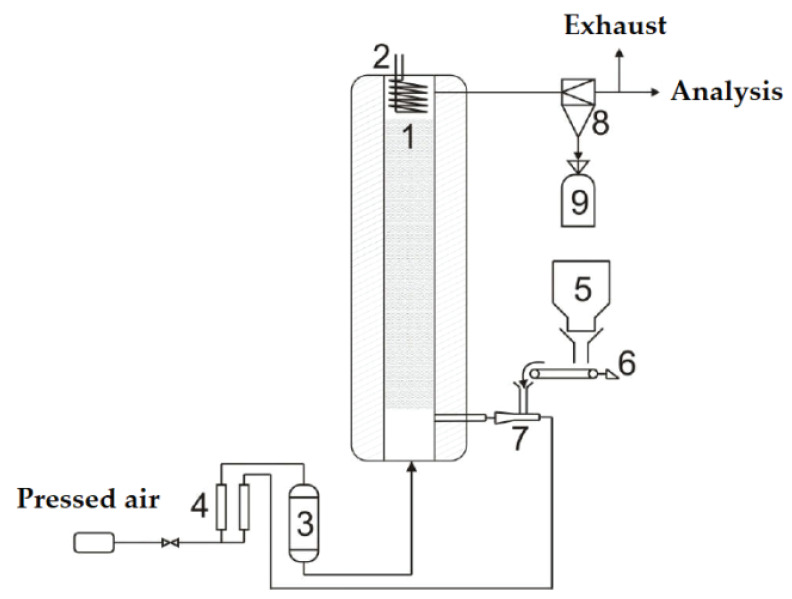
Scheme of the installation for coal combustion in the fluidized bed of a catalyst: 1—reactor; 2—heat exchanger; 3—air heater; 4—rotameters; 5—coal bunker; 6—screw feeder; 7—ejector; 8—cyclone; 9—ash collection bunker.

A scheme of an installation for coal combustion is shown in Figure 2. The installation consists of a fluidized bed reactor with a diameter of 40 mm, of a coal bunker with a screw feeder and an ejector to transfer fuel into the reactor, and of an analysis system.

The preheated air was fed to the reactor bottom to start up the reactor. Airflow rates were controlled with rotameters. Ash particles after the reactor were separated from flue gases in the cyclone and passed into the ash collection bunker. The brown coal combustion was carried out in the fluidized bed of the catalyst with a granule diameter of 1.4–2.0 mm. The loading of the catalyst was 500 mL. The coal combustion was carried out at a constant temperature of 700 °C, an airflow rate of 2.76 m^3^/h, and a coal consumption of 290 g/h (air excess of 3.1). The concentration of pollutants (NO, NO_2_, and CO) in flue gases was determined with a Polar analyzer (Promecopribor Ltd., Moscow, Russia). The degree of burning solid fuel (*β*) was determined by

$$\beta =\frac{10000\left(A-A_{0}\right)}{A\left(100-A_{0}\right)},$$

where *A* is the ash content of dry coal and ash content of remainder after combustion, in percentage. Characteristics of coal are presented in Table 2. The total moisture content of the fuels was determined in accordance with ISO 5068-1:2007. The volatile matter yield was determined in accordance with ISO 5071-1:2013. The ash content in brown coal and product residues was determined in accordance with GOST (State Standard) 55661–2013. The elemental composition of brown coal was determined using a CHNS VARIO EL CUBE elemental analyzer (Elementar Analysensysteme GmbH, Langenselbold, Germany).

## 3. Results and Discussion

### 3.1. Strength and Activity of Spherical CuFeAl-Composite Catalysts

**Figure 3:**
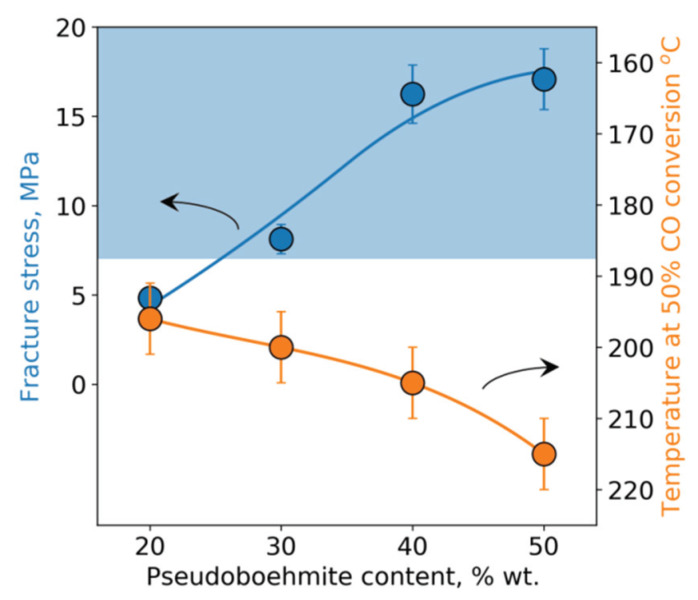
Dependence of the strength (the blue curve and circles) and activity (the orange curve and circles) of the CuFeAl nanocomposite catalyst on the pseudoboehmite content. The blue area is a zone of the required strength.

High mechanical strength and attrition resistance are important characteristics of fluidized bed catalysts. To assess the mechanical strength, the method of measuring the crushing strength of individual granules is usually used as an express method [27]. For the catalysts used in fluidized bed combustion, the minimum fracture stress should be more than 7 MPa [8]. The mechanical interaction of the catalyst granules with such a fracture stress leads mainly to their attrition as a result of mutual friction, and the catalyst loss in this case usually does not exceed 0.5% per day. In contrast, the lower values of fracture stress (<7 MPa) lead to the significant cracking of the catalyst granules during operation, and this leads to an increase in catalyst waste, sometimes by more than 5% per day [2]. One of the main objectives of this work was to study the dependence of the fracture stress and activity of a spherical composite CuFeAl catalyst on the content of pseudoboehmite added at the molding stage (Figure 3). It is important to note that at the pseudoboehmite content of less than 20%, it was not possible to obtain uniform spherical granules of the CuFeAl nanocomposite catalyst.

As seen from Figure 3, an increase in the pseudoboehmite content leads to an increase in the strength of the obtained catalyst granules and to a simultaneous decrease in the activity of the catalyst. The monotonic decrease in activity is associated with a decrease in the content of the active component in the catalyst due to its dilution with alumina, which is known to be inactive in deep oxidation reactions. It is worth mentioning that the activity of all four catalysts with the different pseudoboehmite content exceeds the activity of an industrial catalyst Cu*_x_*Mg_1−*x*_Cr_2_O_4_/Al_2_O_3_ (composition: 1.7% CuO, 3.6% MgO, 17.0% Cr_2_O_3_, and the rest Al_2_O_3_), for which the temperature of the 50%-conversion of CO is 240 °C.

At a pseudoboehmite content of ≥40%, the obtained granules of the spherical CuFeAl nanocomposite catalyst have a high mechanical fracture stress, exceeding an average of 15 MPa. However, it is necessary to take into account the variance of this value [28,29,30]. This is important because porous materials contain defects that are randomly distributed over the volume. When a static force is applied, a granule cracks in the place with the maximum concentration of these defects. The presence of these defects and their random distribution lead to a wide distribution of the values of mechanical strength. The Weibull distribution is commonly used for describing the obtained experimental data [28]. However, in our previous work [31], it was shown that for a statistical description of the mechanical strength of alumina-based materials, it is necessary to use a two-parameter Gamma distribution. Note that the parameter *α* of Gamma distribution determines the distribution width, and the higher *α* is, the narrower the distribution is. The parameters of the Gamma and Weibull distributions were calculated (Table 3) for the obtained CuFeAl nanocomposite catalysts and the industrial Cr-containing catalyst. As seen from the presented data, both the parameter *α* and the Weibull modulus m monotonously decrease with an increase in the pseudoboehmite content, which is accompanied by an increase in the granule strength. The parameter *α* for the Cr-containing catalyst is slightly lower than that for the CuFeAl nanocomposite catalysts. The values of the Weibull modulus m for the studied catalysts are in the range of 3.5–4.4, which is consistent with the published data. Antonyuk et al. [32] obtained the Weibull modulus *m* ≈ 5 for spherical *γ*-Al_2_O_3_ granules. For a series of water gas shift catalysts based on alumina, M. Zakeri et al. [29] observed values of m in the range of 2.5–11.

Based on the obtained Gamma distribution parameters, the fraction of granules with the fracture stress less than 7 MPa was estimated (Table 3). The calculation procedure is described elsewhere [31]. When the pseudoboehmite content achieves 40–50%, the fraction of granules with the low fracture stress decreases to less than 1% (0.6–0.7%). At the same time, in the case of the industrial Cr-containing catalyst, the fraction of fragile granules is about 2.7%. This is due to both the lower strength of the industrial catalyst granules and a lower Gamma distribution parameter *α*. Consequently, it can be expected that the loss of the CuFeAl nanocomposite catalyst during operation in the fluidized bed will be lower than that of the Cr-containing industrial catalyst.

According to the data obtained, it can be concluded that the developed spherical CuFeAl nanocomposite catalyst has high mechanical strength and activity in the oxidation of CO, which are comparable to the characteristics of the industrial Cr-containing catalyst. The catalyst with the 40% pseudoboehmite content was used for further research.

### 3.2. Thermal Stability of Spherical CuFeAl Nanocomposite Catalysts

**Figure 4:**
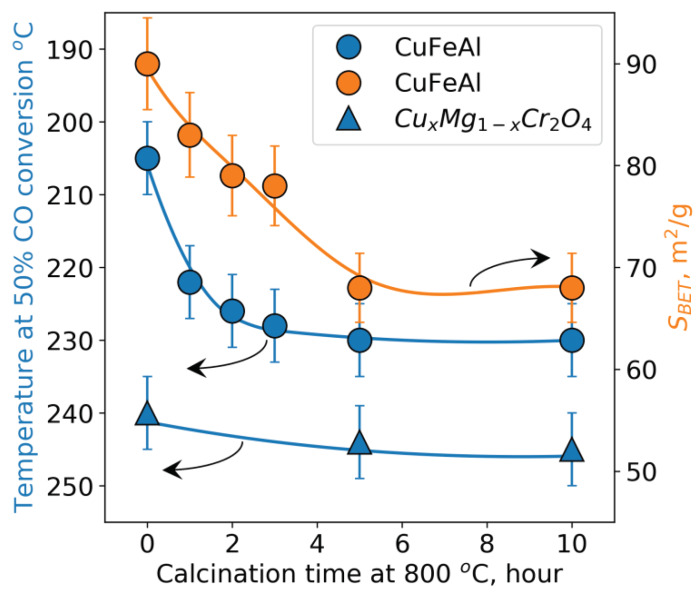
Dependence of activity (the blue curves and circles) and specific surface area (the orange curve and circles) of the studied catalysts on the calcination time at 800 °C, and dependence of activity (the blue curves and triangles) of the commercial catalysts.

As noted above, one of the important requirements for catalysts of deep oxidation in a fluidized bed is their thermal stability [33,34,35]. This requirement arises from the high operating temperature of about 700–750 °C and short-term and/or local overheating up to 1000 °C [2]. Therefore, one of the main goals of this study was to investigate the effect of temperature treatment at 800 °C on the activity and physicochemical properties of the spherical CuFeAl nanocomposite catalyst. Figure 4 shows the dependence of activity and specific surface area of the studied catalysts on the calcination time at a temperature of 800 °C.

As seen from Figure 4, the activity of the spherical CuFeAl nanocomposite catalyst decreases during the first 5 h of calcination and stabilizes after that. Figure 4 also shows that the decrease in surface area is slower than the decrease in activity. It can be assumed that the initial decrease in activity is associated with the sintering of the active component and the excipient (alumina introduced at the oil-drop granulation stage), since the spherical CuFeAl catalyst is a complex composite consisting of the active component and the excipient. A further decrease in the surface is accompanied by the sintering of alumina (excipient), which is not active in deep oxidation reactions. After 5 h, there is no noticeable decrease in activity and specific surface area due to stabilization of the catalyst structure. In addition, the activity of the spherical catalyst calcined at 800 °C is higher than that of the industrial Cr-containing catalyst over the whole range of calcination times (Figure 4).

In the case of the initial powdered catalyst, its activity and specific surface area decrease stronger than those of the spherical catalyst. Table 4 demonstrates the effect of heat treatment on the powdered and spherical catalysts. As seen, the temperature of 50%-conversion of CO for the powdered catalyst increases from 190 to 240 °C (50 °C), while for the spherical catalyst, it increases from 195 to 230 °C (35 °C). One can see that in the case of the spherical catalyst, the decrease in catalytic activity is lower than for the powdered catalyst during the calcination at 800 °C for 5 h (Table 4). Since the spherical catalyst consists of a powdered catalyst and alumina introduced at the oil-drop granulation stage, it could be assumed that alumina stabilizes the active component at high temperatures.

**Figure 5:**
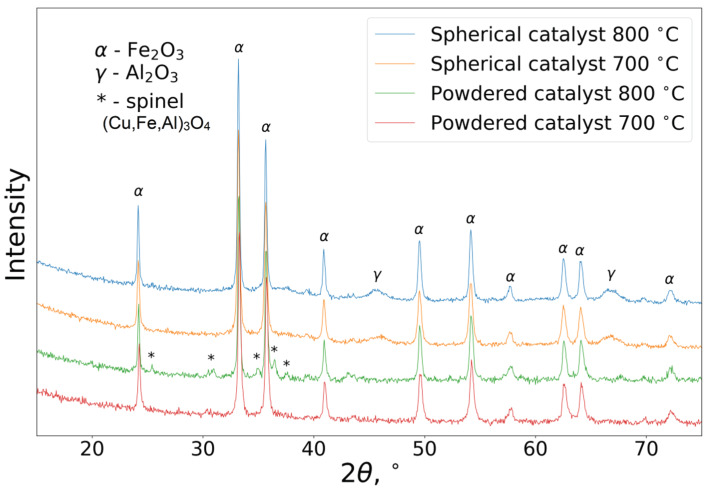
XRD patterns of CuFeAl catalysts.

Figure 5 shows X-ray diffraction (XRD) patterns of the powdered and spherical CuFeAl nanocomposite catalysts calcined at 700 or 800 °C. As seen, the XRD patterns of all samples contain Fe_2_O_3_ hematite reflexes [JCPDS 330664]. The XRD patterns of the spherical catalysts exhibit broad peaks in the 2θ ranges of 45°–48° and 60°–70°. This indicates the presence of *γ*-Al_2_O_3_ [JCPDS 10-0425] formed from pseudoboehmite during calcination. There are no significant differences in the XRD patterns of the spherical catalysts except for an increase in the coherent scattering region (CSR) for hematite, calculated from the 140 reflection (from 58 to 71 nm) for the sample calcined at 800 °C. In contrast, the XRD patterns of the powdered catalysts differ. In the pattern of the sample calcined at 800 °C, there are low-intensity reflections, indicating the formation of spinel (Cu,Al,Fe)_3_O_4_ with the space group Fd3m. In our previous paper [36], it was shown that for the active component with a higher copper content (10 wt% CuO), copper exists mainly in the form of spinel (Cu_0.24_Fe_0.68_Al_0.08_)_3_O_4_ based on iron oxide. This explains its lower activity in comparison with samples containing 5 wt. % CuO, in which copper is in a highly disperse state. Meng-Fei Luo et al. [37] observed the formation of CuAl_2_O_4_ spinel for a catalyst CuO/Al_2_O_3_ obtained by impregnation and calcination at temperatures above 700 °C. The lower CuO content, as well as the lower calcination temperature, lead to the predominant formation of finely disperse copper-containing particles, which determine the higher activity of these catalysts. Thus, the formation of spinel in the active component calcined at 800 °C may cause a decrease in the activity of these catalysts in the oxidation of CO, along with a decrease in the total specific surface.

**Figure 6:**
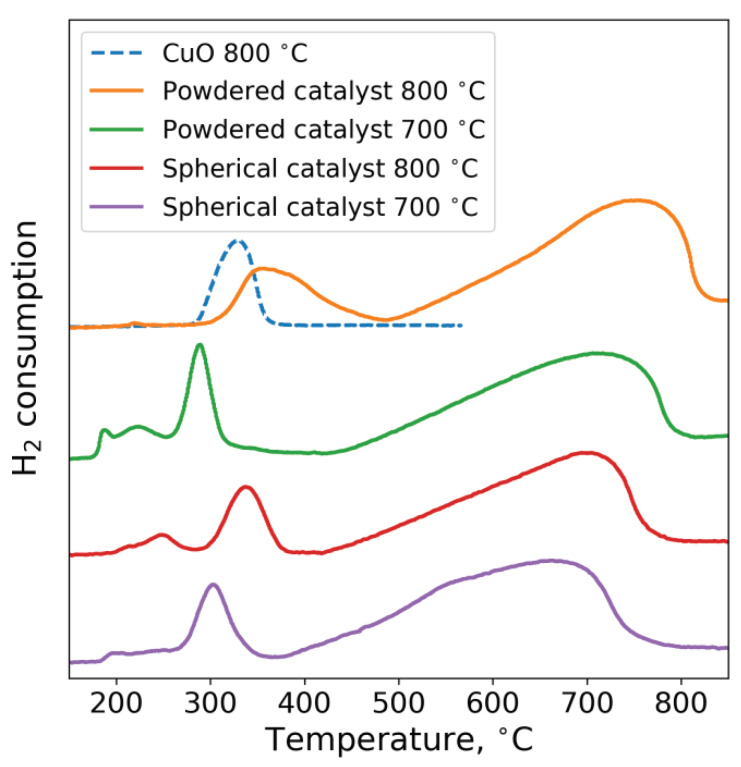
TPR-H_2_ profiles of CuFeAl nanocomposite catalysts.

Figure 6 presents the temperature-programmed hydrogen reduction (TPR-H_2_) curves for the studied catalysts. Curve profiles consist of several peaks. The first peak at temperatures of 200–250 °C is associated with the reduction of copper oxide to the metallic state: CuO→Cu [22,23,37,38]. The peak at 300–350 °C shows the partial reduction of iron(III) oxide: Fe_2_O_3_→Fe_3_O_4_ [22]. The next wide peak at temperatures of 400–900 °C is associated with a step reduction of iron(II,III) oxide to the metallic state: Fe_3_O_4_→FeO→Fe [39]. Note that the increase in calcination temperature does not lead to any significant change in the TPR-H_2_ profile of the spherical catalyst. The only difference is that the reduction peaks of iron oxides shift toward higher temperatures, which is associated with the agglomeration of Fe_2_O_3_ due to calcination. A similar shift was observed by H. Wang [40] during the calcination of CuO-Fe_2_O_3_ catalysts for the NH_3_-SCO reaction. On the contrary, the TPR-H_2_ curve of the powder catalyst changes significantly. For the sample calcined at 800 °C, the low-temperature peak of hydrogen absorption disappears. Moreover, the temperature at which the reduction of this sample starts is higher than that of individual copper oxide calcined under the same conditions. This may be caused by copper being in the form of spinel, which is reduced at a higher temperature and which is less active in the oxidation of CO. The formation of spinel in CuO-Al_2_O_3_ catalysts calcined at temperatures above 700 °C and similar changes in the TPR-H_2_ profiles were reported elsewhere [37,38].

Based on these results, it can be concluded that the developed spherical CuFeAl composite catalyst has high thermal stability. Moreover, the addition of pseudoboehmite at the stage of drop formation not only contributes to obtaining the uniform high-strength spherical catalyst, but also facilitates stabilization of the phase state of the active component. Heat treatment at 800 °C does not lead to the formation of copper-containing phases with the spinel structure, which are less active in the oxidation of CO. Therefore, the detected decrease in activity is mainly caused by a decrease in the total specific surface area as a result of the sintering of the active component.

### 3.3. Coal Combustion in a Fluidized Bed of a Catalyst

The final stage of this work included the production of a batch of the catalyst and its testing in the process of burning brown coal in a fluidized bed. The combustion was carried out for 2 h. The concentration of harmful substances (CO, NO*_x_*, and CH_4_) in the exhaust gases was measured on-line using a gas analyzer with an analysis frequency of 1 Hz. Every 30 min, an ash sample was taken to determine the degree of burnout. Table 5 presents the tests results using three samples: the developed CuFeAl nanocomposite catalyst, the industrial Cr-containing catalyst, and an inert material (quartz sand). The specific surface area for the quartz sand is less than 1 m^2^/g.

For both catalysts, the concentrations of harmful substances and the degree of burnout during the experiment were constant (<5% relative difference). First, it is worth noting that with the use of the catalysts, the degree of burnout achieved 95% (in contrast, only 70% for quartz sand). This is determined by the processes of gasification of solid fuel in a fluidized bed of the catalyst. It is known that coke gasification, as the limiting stage of the combustion process, proceeds on the surface of coal particles along the following main routes:

1. C + O_2_ → CO_2_;
2. 2C + O_2_ → 2CO;
3. C + CO_2_ → 2CO;
4. C + H_2_O → CO + H_2_.

**Figure 7:**
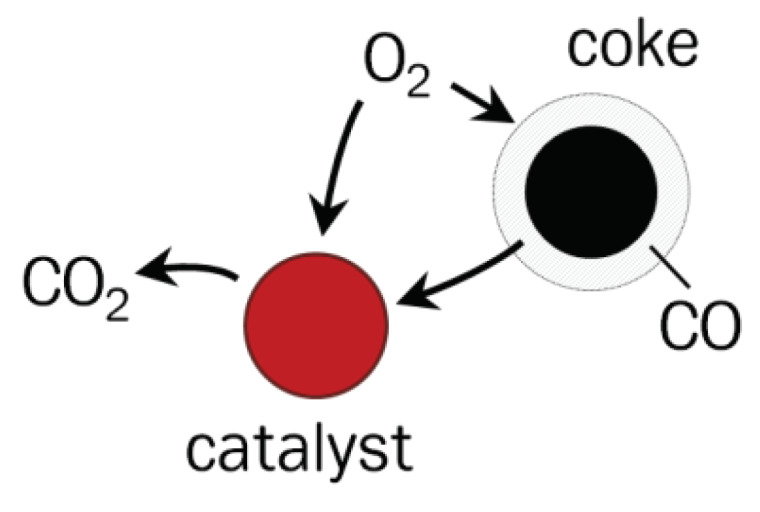
Scheme of coke gasification in a fluidized catalyst bed.

At low combustion temperatures, the coke gasification is limited by the diffusion of oxygen to the surface of coke particles with the formation of CO and CO_2_ [41]. In the case of catalytic deep oxidation, the concentration of CO in the surface layer of the coke particle, and in the gas phase as a whole, is significantly reduced since the catalyst accelerates the oxidation of CO and volatile organic substances (VOC) to the products of complete oxidation (CO_2_ and H_2_O). This significantly increases the diffusion of oxygen to the surface of coke particles and, as a consequence, increases the rate of coke gasification. The combustion in the catalyst bed is schematically shown in Figure 7. It explains the observed increase in the degree of burnout from 70% to 95% and a significant reduction in CO emissions when using the catalysts instead of quartz sand. Moreover, for both the industrial Cr-containing catalyst and the CuFeAl nanocomposite catalyst, the CO emissions are approximately the same and are in the range of 600–700 ppm. The concentration of CH_4_ during the combustion for all samples was below the detection limit of the analyzer (<0.01%).

Nitrogen oxides NO*_x_* (NO and NO_2_) are well known to be among the most common harmful emissions generated during fuel combustion. NO_x_ can be formed both as a result of the oxidation of atmospheric nitrogen by a radical mechanism (thermal NO*_x_*) and as a result of the oxidation of organic fuel compounds containing nitrogen (fuel NO*_x_*). The contribution of the radical oxidation mechanism at temperatures below 750 °C is insignificant, and NO*_x_* is mainly formed as a result of the oxidation of nitrogen-containing fuel components [42]. During the catalytic combustion of brown coal in a fluidized bed, the concentration of NO was near 230–240 ppm. The concentration of NO_2_ for all samples was <10 ppm, which is determined by the thermodynamic equilibrium of the formation of nitrogen oxides (2NO + O_2_ ⇄ 2NO_2_) during fuel combustion [43]. In the case of quartz sand, the NO concentration was half of the value observed for the catalysts (~100 ppm). A lower emission of NO in this case was due to a high concentration of CO and, as a consequence, due to a high contribution from the reactions of the reduction of nitrogen oxides [44]:

5. NO_2_ + CO → NO + CO_2_;
6. NO + 2CO → N_2_ + 2CO_2_.

Thus, the use of the CuFeAl nanocomposite catalyst for the burning of brown coal allows one to achieve low concentrations of CO and NO_x_ and a high degree of fuel burnout, which is close to that for the industrial Cr-containing catalyst. On the basis of the physico-chemical and catalytic characteristics, it can be concluded that the developed spherical CuFeAl nanocomposite catalyst satisfies the basic requirements for deep oxidation catalysts for a fluidized bed and can be recommended for the combustion of gaseous, liquid, and solid fuels.

## 4. Conclusions

A series of spherical CuFeAl nanocomposite catalysts was prepared by the oil-drop granulation method. The samples were investigated by XRD, TPR-H_2_, low-temperature nitrogen adsorption, and crushing strength. Their catalytic properties were studied in the oxidation of CO and burning of brown coal. The burning of coal was studied in a fluidized bed of quartz sand, the spherical CuFeAl nanocomposite, and the industrial Cr-containing catalyst. It was shown that the developed spherical CuFeAl nanocomposite catalyst demonstrates high activity in the CO oxidation, slightly exceeding the characteristics of the industrial Cr-containing catalyst. In addition, the mechanical strength characteristics of the synthesized catalyst (fracture stress of about 16 MPa) comply with the requirements for deep oxidation catalysts for a fluidized bed. The developed catalyst has high thermal stability (up to 800 °C). The addition of pseudoboehmite at the stage of drop formation not only leads to the formation of uniform high-strength granules, but also allows one to stabilize the phase state of the active component. Heat treatment at 800 °C does not lead to the formation of a copper-containing phase with the spinel structure, which is less active in the oxidation of CO. The decrease in activity detected during the first few hours of the reaction is mainly caused by a decrease in the total specific surface area as a result of the sintering of the active component. The use of the CuFeAl nanocomposite catalyst for the burning of brown coal provides low concentrations of CO (600 ppm) and NO*_x_* (220 ppm) as well as a high degree of burnout (95%), which is close to that of the industrial Cr-containing catalyst.

## Figures and Tables

**Table 1:** Conditions used for preparation of the spherical CuFeAl composite catalysts. The data for obtaining 10 g of spherical catalyst.

AlOOH Content, wt%	HNO_3_:Al_2_O_3_ Molar Ratio	H_2_O Content, wt%	Powdered Catalyst, Weight, g	AlOOH Weight, g	2 M HNO_3_, Vol. mL	H_2_O Weight, g
20	0.100	85	8.40	2.10	1.47	56.6
30	0.084	82	7.59	3.24	1.90	45.6
40	0.067	78	6.71	4.47	2.10	35.5
50	0.050	75	5.69	5.69	1.99	30.0

**Table 2:** Physicochemical characteristics of brown coal.

Moisture, %	Ash Content, %	Volatiles, %	C, %	H, %	N, %	O, %	S, %
9.8	11.1	48.0	54.6	4.4	1.5	26.9	1.5

**Table 3:** Catalytic and mechanical properties of the CuFeAl composite and Cr-containing catalysts.

Catalyst	Pseudoboehmite Content, wt%	T_50_, °C	Fracture Stress, MPa	Fraction of Fragile Granules (<7 MPa), %	α Parameter	m, Weibull Modulus
CuFeAl nanocomposite catalysts	20	196	4.3	94	13.4	4.4
	30	200	8.1	35	12.8	4.1
	40	205	16.2	0.7	12.2	3.9
	50	215	17.1	0.6	11.2	3.5
Cu*_x_*Mg_1−*x*_Cr_2_O_4_/Al_2_O_3_	-	240	14.4	2.7	10.0	3.5

**Table 4:** Catalytic and physico-chemical properties of CuFeAl catalysts calcined at different conditions.

Catalyst		Powdered CuFeAl Catalyst		Spherical CuFeAl Catalyst	
Calcination conditions		700 °C 1 h	800°C 5 h	700 °C 1 h	800°C 5 h
T_50_, °C		190	240	195	230
SSA, m^2^/g		60	12	90	68
Composition, wt%	CuO	5.0		3.3	
	Fe_2_O_3_	77.9		51.1	
	Al_2_O_3_	17.1		45.6	

**Table 5:** Catalytic properties of the CuFeAl composite and Cr-containing catalysts.

Catalyst	CO, ppm	NO, ppm	Coal Burnout, %
SiO_2_ (quartz sand)	>8000	100	70
Spherical CuFeAl nanocomposite catalyst *	600	220	95
Cu*_x_*Mg_1−*x*_Cr_2_O_4_/Al_2_O_3_	700	230	95

* the catalyst was calcined at 700 °C for 1 h.
